# Complete chloroplast genome of the marine red alga *Rhodochorton tenue* (Rhodochortonaceae, Rhodophyta) from San Juan Island, Washington

**DOI:** 10.1128/mra.01068-23

**Published:** 2024-01-30

**Authors:** Layla T. Ahmed, Hiba Alesmail, Stephanie Beltran Rodriguez, Rachel Christian, Jonathan Coronado, Alice A. Elledge, America Estrada, Alena Fierro, Angel Garcia Mora, Kayla Gonzalez, Samantha Gonzalez-Leon, Arely M. Guijarro, Jennifer Islas-Quintana, David Juarez-Guido, Jeffery R. Hughey, Edward J. Lara, Jamileth Lara, Carson T. Leonard, Kaylee A. Lockard, Enzou Lopez, Stephanie Martin, Miriam Martinez, Brianna Mederos, Alejandro Medina Pizano, Casey J. Medley, Sarah Mohsin, Thomas F. Mumford, Raphael Araujo Muñoz, Renee Nachtigall, Jannette Noriega, Pedro Ochoa Cendejas, Jessika Ordaz, Alberto J. Parra, Julian Pizano, Michelle Reimold, Kristalyn Rivera, Ayleen Rocha, Karolina C. Rodriguez, Ivan Tena-Garcia, Matthew M. Vargas, Jose Velasquez, On behalf of Hartnell College Genomics Group

**Affiliations:** 1 Division of Mathematics, Science, and Engineering, Hartnell College, Salinas, California, USA; 2 Friday Harbor Laboratories, University of Washington, Friday Harbor, Washington, USA; Department of Biology, Queens College, , USA

**Keywords:** acrochaetiales, chloroplast genome, plastid, *rbc*L, *Rhodochorton purpureum*

## Abstract

We present the complete chloroplast genome sequence of *Rhodochorton tenue* from San Juan Island, Washington. The chloroplast genome of *R. tenue* is 192,037 bp in length, contains 244 genes, and is similar in content to *Acrochaetium secundatum. Rhodochorton tenue* is genetically distinct from *Rhodochorton purpureum* from the North Atlantic Ocean.

## ANNOUNCEMENT

*Rhodochorton tenue* Kylin was originally described as a short plant 3–5 mm high with more or less branched filaments and a creeping basal layer (1). The type locality of *R. tenue* was cited as San Juan Island, Friday Harbor Marine Laboratories Preserve, where it was said to densely cover rocks and stones at the high-water mark (1). Later workers accepted *R. tenue* (2, 3); however, based on similarities in morphology and life histories in culture, West (4) proposed that *R. tenue* be placed into synonymy under the generitype of *Rhodochorton* Nägeli, *Rhodochorton purpureum* (Lightfoot) Rosenvinge. The only two DNA marker sequences of *R. tenue* deposited in GenBank are from slowly evolving (SSU and LSU) genes, and the authors did not comment on the status of the name (5). To determine the systematic relationship between *R. tenue* and *R. purpureum*, the complete chloroplast genome of *R. tenue* was assembled and analyzed.

The specimen of *R. tenue* analyzed here was collected on rocks in the supralittoral from the Friday Harbor Marine Laboratories Preserve, San Juan Island, Washington, voucher number UC 2100479. The DNA was extracted using the DNeasy Blood and Tissue Kit (Qiagen) following a previously published protocol (6). The 150-bp paired-end library was constructed with the KAPA HyperPlus Kit (Roche) and sequenced on an Illumina NovaSeq 6000. The analysis generated 21,656,624 reads that were filtered using the default BBDuk settings in Geneious Prime 2019.1.3 (Biomatters Limited). The chloroplast genome was assembled *de novo* using the filtered reads with a kmer ≥69 in MEGAHIT 1.2.9 (7). The assembly yielded 63,951 contigs with an *N*_50_ of 518 and GC content of 54.6%. Two *R. tenue* chloroplast contigs, 156,855 and 26,268 bp, with 267× and 275× coverage, respectively, were identified by a two-way Nucleotide BLAST search using the default settings. Nuclear and mitochondrial coverages were 659× and 216×, respectively. The final complete chloroplast genome was circularized by iteration over the two gaps using the filtered reads and Map to Reference function with the Low Sensitivity/Fastest setting in Geneious Prime. The genome start position was adjusted to correspond with the chloroplast genome of *Acrochaetium secundatum* (GenBank accession number MH026107). The annotation was performed using the default settings in GeSeq 2.03 (8), followed by manual corrections of gene start and stop positions according to NCBI ORFfinder and Sequin 15.5 (9). Nucleotide identities were calculated by BLAST search using the default settings.

The complete circular chloroplast genome of *R. tenue* is 192,037 bp in length and has a GC content of 33.5%. The genome contains 244 genes including 206 protein-coding, 32 tRNA, and 6 rRNA genes (Table 1). Genome content and structure are highly similar to those of *A. secundatum* (10). The plastid-encoded *rbc*L gene sequence of *R. tenue* differed in nucleotide identity from *R. purpureum* from the Isle of Man, Irish Sea, and Newfoundland and Labrador, Canada, by 1.98%–2.5%. These identities are similar to numbers reported for other florideophyte congeners and support the treatment of *R. tenue* as distinct from *R. purpureum*.

**TABLE 1:** Plastid genome content of *Rhodochorton tenue*

Gene groups	Genes
ATP synthase	atpA, atpB, atpD, atpE, atpF, atpG, atpH, atpI
Cytochrome complex	ccs1, ccsA, petA, petB, petD, petF, petG, petJ, petL, petM, petN
Hypothetical chloroplast orfs	ycf19, ycf20, ycf21, ycf22, ycf23, ycf33, ycf34, ycf35, ycf36, ycf37, ycf39, ycf41, ycf45, ycf46, ycf52, ycf53, ycf54, ycf55, ycf60, ycf65
Maintenance	dnaB, rne, rnz
Metabolism	accA, accB, accD, acpP, argB, carA, cbbX, chlB, chlI, chlL, chlN, dfr, fabH, gltB, ilvB, ilvH, moeB, odpA, odpB, pgmA, preA, rbcL, rbcS, syfB, syh, thiG, thiS, trpA, trpG, upp
Open reading frames	rf55, orf118, orf166, orf257, orf396, orf456, orf493, orf770
Photosystem I	psaA, psaB, psaC, psaD, psaE, psaF, psaI, psaJ, psaK, psaL, psaM, ycf3, ycf4
Photosystem II	psbA, psbB, psbC, psbD, psbE, psbF, psbH, psbI, psbJ, psbK, psbL, psbN, psbT, psbV, psbW, psbX, psbY, psbZ, psb30
Phycobiliproteins	apcA, apcB, apcD, apcE, apcF, cpcA, cpcB, cpcG, cpcS, cpeA, cpeB, nblA
Protein quality control	dnaK, clpC, ftsH, groEL
Redox system	acsF, bas1, dsbD, ftrB, grx, pbsA, trxA
Ribonuclease	rnpB
Ribosomal proteins (large subunit)	rpl1, rpl2, rpl3, rpl4, rpl5, rpl6, rpl9, rpl11, rpl12, rpl13, rpl14, rpl16, rpl18, rpl19, rpl20, rpl21, rpl22, rpl23, rpl24, rpl27, rpl28, rpl29, rpl31, rpl32, rpl33, rpl34, rpl35, rpl36
Ribosomal proteins (small subunit)	rps1, rps2, rps3, rps4, rps5, rps6, rps7, rps8, rps9, rps10, rps11, rps12, rps13, rps14, rps16, rps17, rps18, rps19, rps20
Ribosomal RNAs	rnl(x2), rrn5(x2), rns(x2)
RNA polymerase	rpoA, rpoB, rpoC1, rpoC2, rpoZ
Transcription factors	lysR, ntcA, ompR, ycf29
Transfer RNAs	trnA-TGC(x2), trnC-GCA, trnD-GTC, trnE-TTC, trnF-GAA, trnG-GCC, trnG-TCC, trnH-GTG, trnI-GAT(x2), trnK-TTT, trnL-CAA, trnL-TAA, trnL-TAG, trnM-CAT(x2), trnN-GTT, trnP-TGG, trnQ-TTG, trnR-ACG, trnR-CCG, trnR-TCT, trnS-GCT, trnS-GGA, trnS-TGA, trnT-GGT, trnT-TGT, trnV-GAC, trnV-TAC, trnW-CCA, trnY-GTA
Translation	infB, infC, tsf, tufA
Transport	cemA, secA, secG, secY, sufB, sufC, tatC, ycf38, ycf63
tRNA processing	tilS

## Data Availability

The complete chloroplast genome sequence of *Rhodochorton tenue* is available in GenBank under accession number OR608737. The associated BioProject, SRA, and BioSample numbers are PRJNA1034540, SRS19378060, and SAMN38060163, respectively. The chloroplast genome referenced in the text was *Acrochaetium secundatum* GenBank accession number MH026107.
